# Diagnostic Accuracy of *Mycobacterium tuberculosis* Antigen-Based Skin Tests (TBSTs) for Tuberculosis Infection Compared with TST and IGRA: A Network Meta-Analysis

**DOI:** 10.3390/pathogens13121050

**Published:** 2024-11-29

**Authors:** Li Peng, Weijie Ma, Lei Zhong, Jiaru Yang, Hanxin Wu, Liangyu Zhu, Xun Huang, Rui Yang, Bingxue Li, Weijiang Ma, Xinya Wu, Jieqin Song, Suyi Luo, Fukai Bao, Aihua Liu

**Affiliations:** 1 Yunnan Province Key Laboratory of Children’s Major Diseases Research, School of Basic Medical Sciences, Kunming Medical University, Kunming 650500, China; pengli@kmmu.edu.cn (L.P.); 20220023@kmmu.edu.cn (W.M.); 20220022@kmmu.edu.cn (L.Z.); jiaru.yang1@monash.edu (J.Y.); 20221746@kmmu.edu.cn (H.W.); 20231847@kmmu.edu.cn (L.Z.); 20230028@kmmu.edu.cn (X.H.); 20230029@kmmu.edu.cn (R.Y.); libingxue1@kmmu.edu.cn (B.L.); 20221745@kmmu.edu.cn (W.M.); 20210037@kmmu.edu.cn (X.W.); 20211707@kmmu.edu.cn (J.S.); luosuyi@kmmu.edu.cn (S.L.); 2 Research Center, Baoshan People’s Hospital, Baoshan 678000, China; 3 Yunnan Provincial Key Laboratory of Public Health and Biosafety, School of Public Health, Kunming Medical University, Kunming 650500, China

**Keywords:** tuberculosis, diagnosis, TST, IGRA, the *Mycobacterium tuberculosis* antigen-based skin test, meta-analysis, diagnostic test accuracy

## Abstract

The aim of this study was to evaluate the diagnostic accuracy of the IGRA, TST, and TBST by combining diagnostic test accuracy (DTA) analysis and network meta-analysis (NMA) to increase the reliability and accuracy of diagnostic methods and promote the eradication of TB. An electronic search of the PubMed, Embase, and Cochrane databases was conducted, from the date of establishment to September 30, 2024. Data were synthesized with frequentist random-effects network meta-analyses, a single-group rate meta-analysis algorithm, and a bivariate mixed-effects logistic regression model. Summarized receiver operating characteristic curves and Fagan nomograms were used to assess diagnostic accuracy and clinical utility. Deeks’ funnel plots and the Quality Assessment of Diagnostic Accuracy Studies 2 tools were used to assess publication bias and risk of bias. Sources of heterogeneity were investigated using subgroup analyses. Forty-nine studies were identified. The diagnostic performance of the three diagnostic methods for TB infection is summarized as follows: the pooled sensitivity was 77.9% (95% confidence interval [CI], 0.69–0.856), and the pooled specificity was 80.3% (95% CI, 0.75–0.86). The sensitivity and specificity of the IGRA were 82.1% (95% CI, 0.78–0.86) and 81.1% (95% CI, 0.75–0.86), respectively, both higher than the TST. However, the TBST exhibited the highest specificity, at 98.5% (95% CI, 0.96–1.00), with a sensitivity of 78.7% (95% CI, 0.68–0.88), which was between that of the IGRA and TST. Subgroup analysis found that population categories and reference standards, among other factors, may be attributed to heterogeneity. In addition, the TST and IGRA add-on TBST can significantly improve diagnostic accuracy. In our study, the IGRA showed higher sensitivity, whereas the TBST showed higher specificity. Interestingly, under certain conditions, TST add-on TBST and IGRA add-on TBST showed better accuracy than TST and IGRA alone and could provide more effective guidance for clinical practice (PROSPERO CRD42023420136).

## 1. Introduction

Tuberculosis (TB), a human disease caused by *Mycobacterium tuberculosis* (*Mtb*), is a major challenge facing global public health. The Global Tuberculosis Report 2023 showed that 47 countries had achieved or exceeded the first phase of the End TB Epidemic strategy by 2022, but TB remains the world’s second leading cause of death from a single infectious source, after severe acute respiratory syndrome coronavirus 2 infection, causing almost twice as many deaths as acquired immunodeficiency syndrome [1]. Most recent studies have considered culturing of Mtb as the gold standard for diagnosing TB infection [2,3,4], but culturing Mtb is time-consuming, which is not conducive to the early diagnosis of TB infection and subsequent implementation of treatment measures; therefore, more sensitive and effective methods to detect and diagnose TB infection are needed.

The tuberculin skin test (TST) is a subcutaneous injection of a purified protein derivative secreted by *Mtb* into the forearm of the patient, which induces a T lymphocyte-mediated delayed hypersensitivity reaction; the test is considered positive or negative based on the size of the induration [5,6]. The interferon-gamma release assay (IGRA) is an in vitro blood test for T cell-mediated immune responses to *Mtb*-specific antigens, early secretory antigenic target 6 kDa protein (ESAT-6), and culture filtrate protein 10 (CFP-10). There are two main categories of IGRAs. The first category includes enzyme-linked immunosorbent assays that detect interferon-γ released in response to ESAT-6 and CFP-10 stimulation, such as the QuantiFERON-TB Gold Plus (QFT-Plus) and WANTAI TB-IGRA tests, depending on the specific antigen used. The second category of IGRAs, which includes the Oxford Immunotec T-SPOT^®^. *TB* assays (T-SPOT.TB), quantify the number of T lymphocytes that produce interferon-γ following stimulation of peripheral blood mononuclear cells with ESAT-6 and CFP-10 [6,7]. The *Mycobacterium tuberculosis* antigen-based skin test (TBST), a newer method for detecting TB infection and recommended by the World Health Organization in 2021 [8], is divided into three categories according to specific recombinant antigens (ESAT-6 or CFP-10) and dosage: C-Tb/Cy-Tb (Serum Institute of India), Diaskintest (Generium, Russian Federation), and Creation-TST (C-TST, China) [9]. However, current diagnostic methods for TB infection (TST, IGRA, and TBST) have a variety of limitations [10,11]. The use of non-specific *Mtb* antigens in the TST may produce non-specific reactions, increasing the likelihood of false-positive and false-negative results. The IGRA cannot distinguish between active tuberculosis and latent tuberculosis infection, and the TBST is limited by the same problem. Selecting a method that provides high diagnostic accuracy is thus crucial for the accurate diagnosis of TB infection. Therefore, our present study compared the accuracy of the TST, IGRA, and TBST for the diagnosis of TB infection in order to provide better guidance for the clinical selection of methods to diagnose TB infection.

Diagnostic test accuracy (DTA) is a type of systematic review recommended by Cochrane to comprehensively assess the accuracy of a diagnostic test and enable clinicians to make better healthcare decisions based on the latest research evidence [12]. Network meta-analysis (NMA) refers to a type of meta-analysis based on a weighted pooled analysis of all studies in a body of evidence consisting of three or more interventions, combined with direct and indirect comparisons [13]. Our present study comprehensively evaluated the accuracy of the TST, IGRA, and TBST for detecting TB infection using a combined DTA analysis and NMA approach. The detection methods were compared in order to obtain a more reliable and accurate diagnostic method, provide better guidance for the selection of clinical diagnosis methods for TB infection, and thereby help facilitate the eventual eradication of TB.

## 2. Methods

This diagnostic accuracy NMA was conducted according to criteria reported by the Preferred Reporting Items for Systematic Reviews and Meta-Analyses (PRISMA) of DTA studies [14]. Studies that assessed the diagnostic accuracy of the TBST, TST, or IGRA were eligible for this review. The protocol of the network meta-analysis was registered in PROSPERO (CRD42023420136).

### 2.1. Search Strategy and Literature Inclusion Criteria

Three databases (PubMed, Embase, and Cochrane) were systematically searched from the date of establishment to 30 September 2024 by two researchers (LP and LZ). No restrictions or filters were imposed on the search strategy except human research and English language. The search used the following keywords: ((C-TST OR Diaskintest OR C-Tb OR Cy-Tb) OR (“Interferon-gamma Release Tests” OR “tuberculin test”)) AND Diagnosis AND (tuberculosis OR “*Mycobacterium tuberculosis*”). The detailed search strategy is summarized in Supplementary Table S1.

A systematic literature search was conducted based on the population, intervention, comparator, and outcome principle: (1) population: tuberculosis patients or people infected with *Mtb*; (2) interventions: TBST, TST, or IGRA; (3) comparison: gold standard or reference standard; (4) outcome: sensitivity and specificity or the number of true-positive (TP), true-negative (TN), false-positive (FP), and false-negative (FN) cases. Inclusion criteria for the study were as follows: (1) all diagnostic studies in which patients had any index tests, such as TBST, TST, or IGRA; and (2) publications written in the English language. The exclusion criteria were as follows: (1) case reports; (2) non-human studies; (3) non-original studies (letters, reviews, and editorials); and (4) studies without sufficient information to determine TP, FP, FN, and TN values. References were managed using EndNote version X9.1. After removal of duplicates, the remaining articles were screened and assessed independently by three researchers (LP, LZ, and WJM) based on the title, abstract, and full text. Any disagreements regarding the eligibility of studies for inclusion were addressed via discussion with a third reviewer (KBF or AHL) until a consensus was reached.

### 2.2. Data Extraction

Three researchers (LP, LZ, and WJM) independently extracted the following information from the included studies: first author’s name; publication year and country; study type (prospective, retrospective, or indetermination); gold/reference standard; basic information regarding the research population; diagnostic method; sensitivity, specificity, TP, TN, FP, and FN values for the main outcomes. In case of unreported TP, TN, FP, or FN values, these parameters were calculated from known variables (specificity and sensitivity). Disagreements were resolved by consensus with the co-authors.

### 2.3. Assessment of Risk of Bias

The risk of bias of the included studies was independently assessed using the revised Quality Assessment of Diagnostic Accuracy Studies (QUADAS-2) tool by two researchers (LP and WJM) [15]. This tool assesses the bias of DTA studies in four key domains: patient selection, index test, reference standard, and flow/timing areas. We generated a risk of bias summary and graph using R 4.3.2 software. Discrepancies were resolved through consensus in conjunction with the corresponding author.

### 2.4. Statistical Analysis

To assess the diagnostic accuracy of the TBST, we performed a DTA NMA of three diagnostic methods: TBST, TST, and IRGA. This approach allowed us to make direct and indirect comparisons through a common comparator (i.e., gold/reference standard) [16].

STATA 17.0 (STATA Corp.) was used for analyses with the “networkplot” command to conduct a population analysis of the included studies based on diagnostic methods and sample sources, and a network structure diagram was drawn based on the results. Nodes represented different diagnostic methods and sample sources, and edges represented head-to-head comparisons. The size of the nodes was related to the number of studies. The thickness of the connecting lines represented the number of comparisons between the two tests. We then used the “metaprop” command to calculate the sensitivity, specificity, and respective pooled values for each study and drew forest plots of the results. I^2^ was calculated to indicate heterogeneity [17]; generally, when I^2^ is greater than 50%, the heterogeneity is considered to be high, and when I^2^ is greater than 75%, the heterogeneity is considered to be too high, in which case subgroup analysis is adopted to test the source of heterogeneity. Furthermore, a bivariate random-effects logistic regression model was used to calculate the consolidated statistics (sensitivity, specificity, positive likelihood ratio [PLR], negative likelihood ratio [NLR], and diagnostic odds ratio [DOR]) using “midas”, and a summarized receiver operating characteristic curve (SROC) was plotted to better reflect the direct synergistic changes in sensitivity and specificity [18]. The closer the area under the curve (AUC) value is to 1, the higher the accuracy of the diagnostic test. In addition, Deeks’ funnel-plot asymmetry test was used to evaluate the presence of publication bias [19].

Subgroup analysis: We conducted a subgroup analysis of the three diagnostic methods and the population receiving the bacille Calmette–Guerin (BCG) vaccine and explored the diagnostic accuracy of the TST add-on TBST and IGRA add-on TBST. To better evaluate the diagnostic performance, a Fagan nomogram was plotted to display the relationship between pre-test probability, likelihood ratio (LR), and post-test probability.

## 3. Results

**Figure 1:**
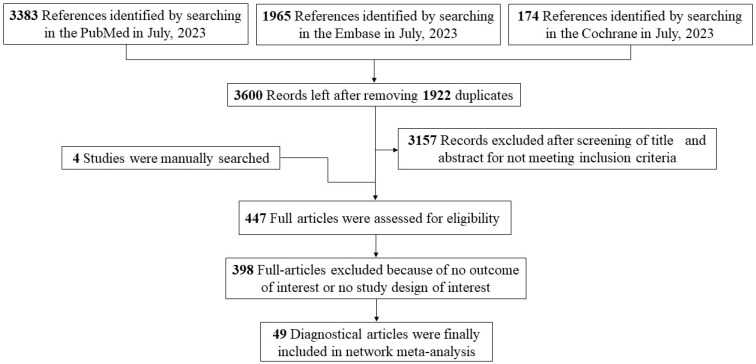
PRISMA flow diagram.

A total of 5522 studies were identified after searching the three databases, with 3383 articles from PubMed, 1965 from Embase, and 174 from Cochrane. After excluding 1922 duplicates, the remaining studies were screened based on title and abstract, and 3157 records that did not meet the inclusion criteria were excluded. A further manual search identified four additional studies. Of 447 full-text articles, 49 met the inclusion criteria and were included in the NMA. The overall workflow is shown in the PRISMA flow diagram below (Figure 1).

The included studies involved 11,402 patients (case numbers ranged from 20 to 1003). Forty studies assessed the performance of the IGRA, fifteen assessed the performance of the TST, and five assessed the performance of the TBST, with 11 studies conducting head-to-head analyses that assessed both the IGRA and the TST. All included subjects were recruited and completed their surveys during the period 2002–2018, and the studies were conducted in China, Japan, Korea, Thailand, Germany, Brazil, the United States of America (USA), and Spain. The majority of studies (42, 86%) were prospective, whereas the remaining studies (7, 14%) were retrospective. Forty-five studies collected blood samples, six collected pleural fluid, two collected alveolar lavage fluid, and one collected synovial mononuclear fluid. The general characteristics of the included studies are shown in the baseline data table (Table 1).

**Figure 2:**
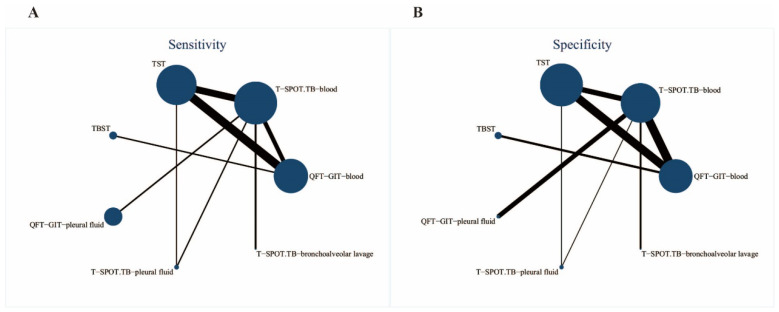
NMA graphs of sensitivity (**A**) and specificity (**B**). Nodes represent different diagnostic methods and sample sources, and connecting lines represent head-to-head comparisons. The size of the node is related to the number of studies. The thickness of the connecting lines indicates a greater number of comparisons between the two tests.

Based on different diagnostic tests for TB infection and different sample sources, the included studies were divided into seven subgroups for pairwise comparisons, and the network diagram of these pairwise comparisons is shown in Figure 2. The larger the size of the node and the thicker its connecting lines, the more studies that were directly compared are indicated. Of the studies included in the NMA, 15 were head-to-head studies, and 9 were three-way head-to-head studies. In the sensitivity analysis (Figure 2A), most of the studies were compared with T-SPOT. TB-blood and the pairwise comparison group of TST and QFT-GIT-blood was also the largest. In the specificity study (Figure 2B), most studies were compared with TST, and more studies compared the pair TST and QFT-GIT-blood.

**Figure 3:**
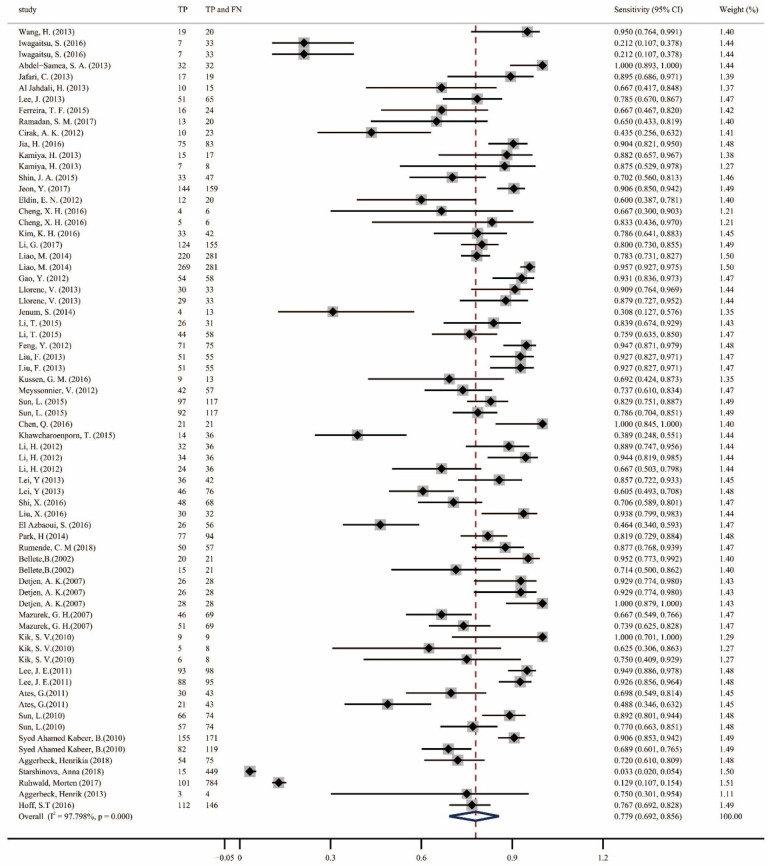
Forest plots of pooled sensitivity for all included studies. TP, true positive; FN, false negative [20,21,22,23,24,25,26,27,28,29,30,31,32,33,34,35,36,37,38,39,40,41,42,43,44,45,46,47,48,49,50,51,52,53,54,55,56,57,58,59,60,61,62,63,64,65,66,67,68].

**Figure 4:**
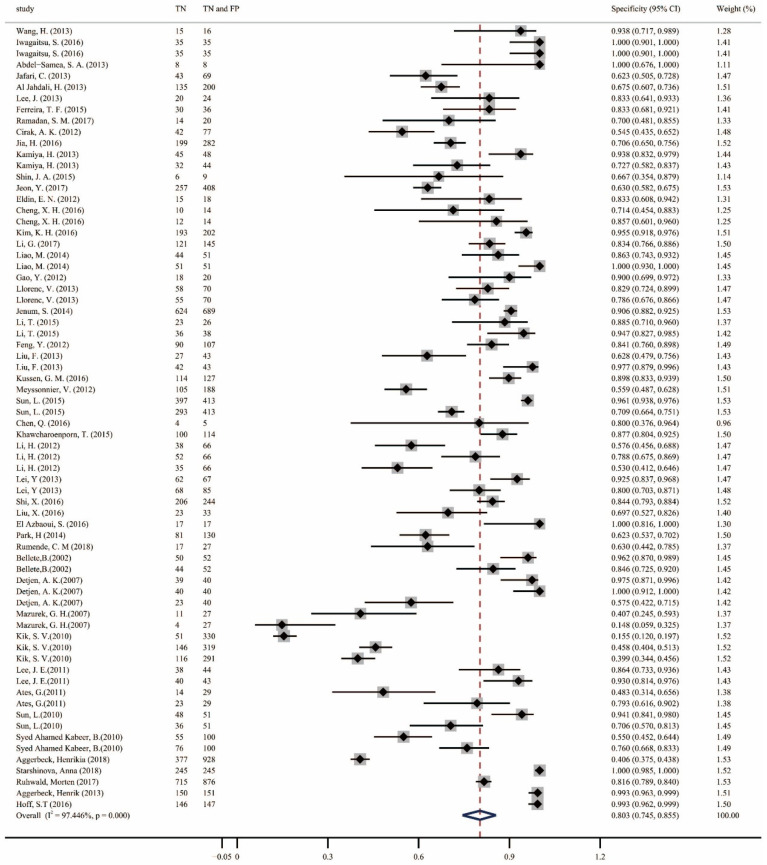
Forest plots of pooled specificity for all included studies. TN, true negative; FP, false positive [20,21,22,23,24,25,26,27,28,29,30,31,32,33,34,35,36,37,38,39,40,41,42,43,44,45,46,47,48,49,50,51,52,53,54,55,56,57,58,59,60,61,62,63,64,65,66,67,68].

A total of 70 datasets were obtained from the 49 included studies. When combining all studies on IGRA, TST, and TBST, the pooled sensitivity was 77.9% (95% confidence interval [CI], 0.69–0.86; Figure 3), and the specificity was 80.3% (95% CI, 0.75–0.86; Figure 4). Forest plots of the three different diagnostic tests for TB infection are shown in the Supplementary Materials (Supplementary Figures S1–S4), and these plots show the included studies’ sensitivities and specificities, as well as the TP, FP, TN, and FN values. The sensitivities of the IGRA, TST, and TBST were 82.1% (95% CI, 0.78–0.86; Supplementary Figure S1), 75.5% (95% CI, 0.65–0.85; Supplementary Figure S3A), and 78.7% (95% CI, 0.68–0.88; Supplementary Figure S4A), respectively, and the specificities were 81.1% (95% CI, 0.75–0.86; Supplementary Figure S2), 73% (95% CI, 0.57–0.87; Supplementary Figure S3B), and 98.5% (95% CI, 0.96–1.0; Supplementary Figure S4B), respectively.

**Figure 5:**
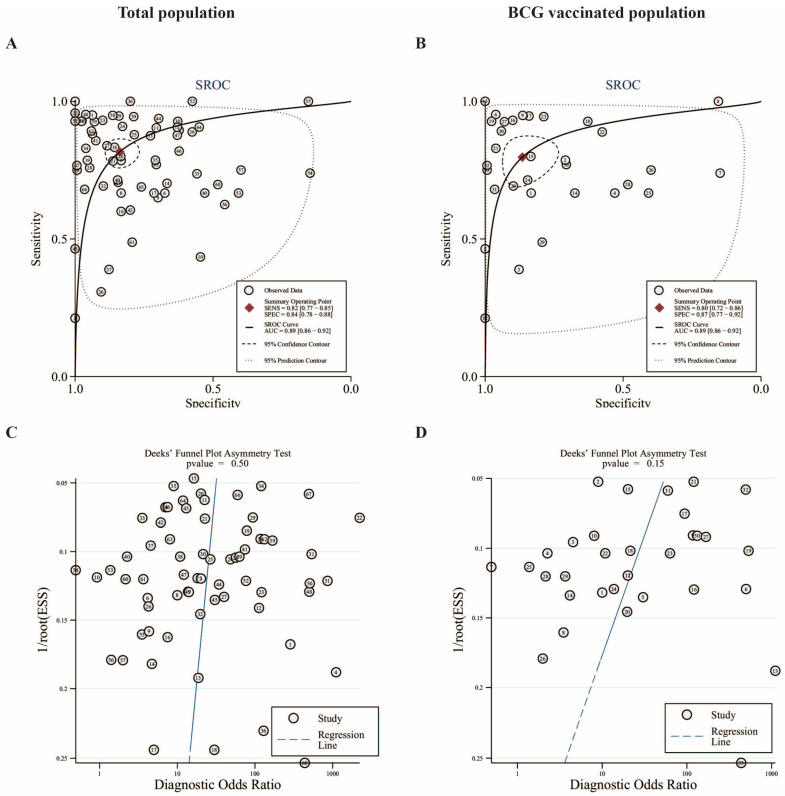
SROC curves and Deeks’ funnel plots for different populations. The total population (**A**), the BCG-vaccinated population (**B**), and the Deeks’ funnel plots for the total population (**C**) and the BCG-vaccinated population (**D**). Curves are shown as straight lines; each study is shown as a circle; summary operating point estimates corresponding to total sensitivity and specificity are shown as red squares; the 95% confidence intervals are shown as dotted lines. SENS: sensitivity; SPEC: specificity.

Based on the included studies, we calculated consolidated statistics and conducted subgroup analyses, plotted SROC curves, and constructed Fagan nomograms to evaluate the diagnostic accuracy of the three diagnostic tests for TB infection. Finally, Deeks’ funnel plots were generated to evaluate publication bias. The pooled AUC of the three diagnostic tests was 0.89 (95% CI, 0.86–0.92); the sensitivity and specificity were 0.82 (95% CI, 0.77–0.85) and 0.84 (95% CI, 0.78–0.88), respectively; and the PLR, NLR, and DOR were 5.1 (95% CI, 3.7–6.8), 0.22 (95% CI, 0.17–0.28), and 23 (95% CI, 15–36), respectively, as shown in Figure 5A and Supplementary Table S2A.

The studies were then screened in the subgroup analysis to identify those that included BCG vaccination, which returned an AUC of 0.89 (95% CI, 0.86–0.92); a sensitivity and specificity of 0.80 (95% CI, 0.72–0.86) and 0.87 (95% CI, 0.77–0.92), respectively; and PLR, NLR, and DOR values of 5.9 (95% CI, 3.4–10.2), 0.23 (95% CI, 0.17–0.33), and 25 (95% CI, 12–51), respectively, as shown in Figure 5B and Supplementary Table S2A. Figure 5C shows the results of the Deeks’ funnel-plot asymmetry test (*p* = 0.50) at the threshold that could produce publication bias. Relatively, the BCG-vaccinated population (*p* = 0.15) was associated with publication bias.

Subgroup analyses were performed using different methods. However, SROC curves could not be plotted separately for the TBST because of insufficient available data (only three of the five included studies contained valid data). In studies that included the total population, the results of the Deeks’ funnel-plot asymmetry test of studies that examined both the IGRA (*p* = 0.67, Supplementary Figure S7C) and TST (*p* = 0.64, Supplementary Figure S7D) did not exhibit publication bias. The AUC for the IGRA was 0.90 (95% CI, 0.87–0.92), and the sensitivity and specificity were 0.83 (95% CI, 0.78–0.87) and 0.83 (95% CI, 0.78–0.88), respectively, whereas the PLR, NLR, and DOR were 5 (95% CI, 3.7–6.9), 0.2 (95% CI, 0.15–0.26), and 25 (95% CI, 15–42), respectively, as shown in Supplementary Figure S7A and Supplementary Table S2B. The AUC for the TST was 0.83 (95% CI, 0.80–0.86), and the sensitivity and specificity were 0.78 (95% CI, 0.64–0.87) and 0.75 (95% CI, 0.60–0.86), respectively, whereas the PLR, NLR, and DOR were 3.1 (95% CI, 1.9–5.2), 0.3 (95% CI, 0.18–0.49), and 11 (95% CI, 5–23), respectively, as shown in Supplementary Figure S7B and Supplementary Table S2B.

As the three TBST studies included the BCG-vaccinated population, we further analyzed the diagnostic performance of TBST add-on TST or IGRA in the BCG-vaccinated population. The AUC of the TST was 0.83 (95% CI, 0.79–0.86); the sensitivity and specificity were 0.77 (95% CI, 0.62–0.88) and 0.74 (95% CI, 0.49–0.90), respectively; and the PLR, NLR, and DOR were 3 (95% CI, 1.3–6.8), 0.3 (95% CI, 0.17–0.54), and 10 (95% CI, 3–33), respectively, as shown in Supplementary Figure S5A and Supplementary Table S2C. The AUC for the TST add-on TBST was 0.85 (95% CI, 0.82–0.88); the sensitivity and specificity were 0.76 (95% CI, 0.64–0.84) and 0.84 (95% CI, 0.66–0.96), respectively; and the PLR, NLR, and DOR were 5.9 (95% CI, 2.0–17.1), 0.28 (95% CI, 0.18–0.42), and 21 (95% CI, 6–74), respectively, as shown in Supplementary Figure S5B and Supplementary Table S2C. The Deeks’ funnel-plot asymmetry test (p = 0.89) of the TST alone did not reveal any publication bias. However, studies of the TST add-on TBST (*p* = 0.48) exhibited publication bias, as shown in Supplementary Figure S5C,D. Furthermore, publication bias in studies using either the IGRA (*p* = 0.14) alone or TBST add-on IGRA (*p* = 0.08) is summarized in Supplementary Figure S9C,D. The AUC for the IGRA was 0.90 (95% CI, 0.88–0.93); the sensitivity and specificity were 0.82 (95% CI, 0.71–0.89) and 0.86 (95% CI, 0.75–0.92), respectively; and the PLR, NLR, and DOR were 5.7 (95% CI, 3.2–10.0), 0.21 (95% CI, 0.13–0.35), and 26 (95% CI, 11–61), respectively, as shown in Supplementary Figure S9A and Supplementary Table S2D. The AUC for IGRA add-on TBST was 0.92 (95% CI, 0.89–0.94); the sensitivity and specificity were 0.81 (95% CI, 0.71–0.88) and 0.9 (95% CI, 0.81–0.95), respectively; and the PLR, NLR, and DOR were 8 (95% CI, 4.2–15.2), 0.22 (95% CI, 0.14–0.33), and 37 (95% CI, 16–84), respectively, as shown in Supplementary Figure S9B and Supplementary Table S2D.

**Figure 6:**
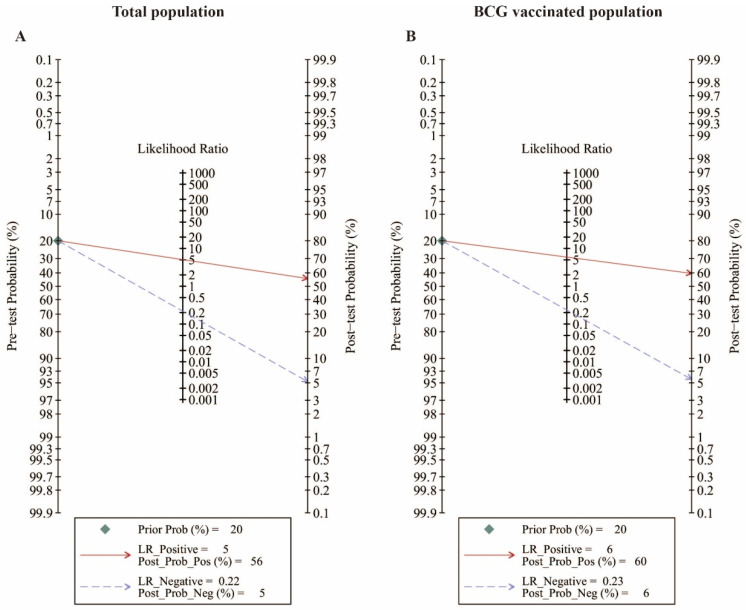
Fagan nomograms for different populations. The total population (**A**) and the BCG-vaccinated population (**B**).

At a pre-test probability of 20%, the post-test probabilities of a positive test result were 56% (total population) and 60% (BCG-vaccinated population), whereas the LR values of 0.22 (total population) and 0.23 (BCG-vaccinated population) reduced the post-test probabilities of a negative test result to 5% (total population) and 6% (BCG-vaccinated population), as shown in Figure 6A,B. In the subsequent subgroup analysis, the post-test probabilities of a positive test result were 56% (IGRA) and 44% (TST), whereas the LR values of 0.20 (IGRA) and 0.30 (TST) reduced the post-test probabilities of a negative test result to 5% (IGRA) and 7% (TST), as shown in Supplementary Figure S8A,B. Finally, the TBST add-on TST and IGRA were analyzed in the BCG-vaccinated population. The post-test probabilities of a positive test result were 43% (TST) and 60% (TST add-on TBST), whereas the LR values of 0.30 (TST) and 0.28 (TST add-on TBST) reduced the post-test probabilities of a negative test result to 7% (TST) and 6% (TST add-on TBST), as shown in Supplementary Figure S6A,B. The post-test probabilities of a positive test result were 59% (IGRA) and 67% (IGRA add-on TBST), whereas the LR values of 0.21 (IGRA) and 0.22 (IGRA add-on TBST) reduced the post-test probabilities of a negative test result to 5% (IGRA) and 5% (IGRA add-on TBST), as shown in Supplementary Figure S10A,B.

The results of our QUADAS-2 assessment are summarized in Table 2 and Supplementary Figure S11. The results showed that most of the studies (n = 47, 96.0%) exhibited a low risk of bias overall. The index text, reference standard, and flow and timing also exhibited low risk of bias.

## 4. Discussion

To our knowledge, this study is the first to compare the diagnostic accuracy of the new TB infection diagnostic method TBST with that of the TST and IGRA. We used two algorithms to analyze the pooled sensitivity and specificity of all the included articles. First, we used the “metaprop” command, a single-group rate meta-analysis algorithm based on a random-effects model, to calculate the pooled sensitivity and specificity, which were 77.9% (95% CI, 0.692–0.856) and 80.3% (95% CI, 0.745–0.855), respectively. The I^2^ values indicated significant heterogeneity, which was also evident from forest plots, which indicated that some studies had extremely low sensitivities and specificities [21,40,57,58,61,62]; this may be one of the reasons for the high heterogeneity. Subsequently, we used the “midas” command based on a bivariate mixed-effects logistic regression model to calculate the consolidated statistics of the included studies. The pooled sensitivity and specificity values were similar to those obtained using the previous algorithm, with values of 0.82 (95% CI, 0.77–0.85) and 0.84 (95% CI, 0.78–0.88), respectively. Deeks’ funnel plots suggested the possibility of publication bias. To identify the causes of heterogeneity and publication bias, as well as to compare the diagnostic accuracy of the three diagnostic methods, we conducted a subgroup analysis and plotted Fagan nomograms. Both the forest plots and Fagan nomograms, as well as the AUCs of the SROC curves, indicate the following: (1) The IGRA provides higher sensitivity, specificity, and diagnostic accuracy than the TST, which is consistent with other research [69,70,71]. (2) Compared with the TST and IGRA, the TBST provides the highest specificity and intermediate sensitivity, indicating that use of the TBST can reduce the possibility of misdiagnosis and false-positive results and enable physicians to more accurately diagnose and evaluate disease state while avoiding unnecessary interventions and treatments, thereby reducing unnecessary use of medical resources. Due to the limited number of TBST studies included, it was not possible to draw SROC curves and compare the synergistic changes in sensitivity and specificity. (3) The combination of the TBST and TST provides improved specificity and diagnostic accuracy while maintaining sensitivity. (4) Similarly, the combination of the TBST and IGRA maintains sensitivity while improving specificity and diagnostic accuracy. These two interesting findings suggest that the TST add-on TBST or IGRA add-on TBST could possibly be used to ensure optimal diagnostic accuracy for TB infection in the future.

We also analyzed the practicality of three diagnostic methods in clinical practice. Fagan nomograms can be used to determine the post-test probability after undergoing a diagnostic test based on the pre-test probability and LR. The pre-test probability refers to the probability of the patient’s illness being present before conducting the diagnostic test or examination. The pre-test probability may vary for different patients under different circumstances. Physicians estimate the pre-test probability based on the patient’s medical history, physical examination, and other factors. Some reports have indicated that the pre-test probability of high clinical suspicion of tuberculosis is 75%, whereas that of low suspicion is 25% [72], but this population classification method does not apply to the studies we included. Despite multiple attempts by researchers to quantify this parameter, there is no consensus regarding the optimal method for determining a therapeutic threshold [73,74,75,76,77]. Boyles et al. used Bayes’ theorem to recalculate the therapeutic threshold. At a post-test probability of 2% and LR of 0.1, they calculated the pre-test probability (equated to the therapeutic threshold in this example) as 20%. This is reasonable, because any test with a pre-test probability greater than 20% will result in a negative test with a post-test probability above 2%, and treatment should be started regardless of the outcome [78]. Therefore, a 20% pre-test probability was used to evaluate the three diagnostic methods in our study. The results indicated that (1) the IGRA can provide more reliable diagnostic information for clinicians and greater diagnostic accuracy than the TST and that (2) after TST add-on TBST and IGRA add-on TBST, the PLR significantly increased, the NLR decreased, and the post-test probability significantly increased, from 43% to 60% and 59% to 67%, respectively. In other words, when the diagnostic result is positive, the probability of the patient actually becoming ill increases, whereas when the diagnostic result is negative, the reliability of the actual diagnosis also increases. It follows that, if conditions permit, both the TST and IGRA add-on with the new diagnostic method TBST can increase the reliability of the diagnosis and provide effective guidance for clinical practice, early detection, early prevention and control, and early treatment, which is conducive to facilitating the eradication of TB.

There are several notable advantages to our systematic review and NMA. First, we used a new statistical method for the NMA developed for DTA studies. Second, we comprehensively evaluated the diagnostic performance of the TBST, TST, and IGRA and studied the diagnostic accuracy of the TBST add-on TST or IGRA in diagnosing TB infection, which greatly improved the diagnostic efficiency. Third, we searched several authoritative medical literature databases, including studies from China, Japan, South Korea, Thailand, Germany, Brazil, the USA, Spain, and other countries, so the conclusions are highly applicable. Fourth, we used a specially designed QUADAS-2 tool to assess the risk of bias, and the risk of bias was low, indicating that our findings are credible. Fifth, we also performed predictive assessments to reflect the diagnostic value of the TBST, TST, and IGRA in TB infection.

## 5. Limitations

Our study had four main limitations. First, the overall heterogeneity of the included studies was not ideal, and the source of heterogeneity was still not identified after subgroup analysis. One potential reason for this result is that the included subjects were from different populations, and the diseases that affect different populations are not consistent. In addition, most of the included articles used clinical diagnosis as the gold standard for the diagnosis of TB infection, which may also be due to inconsistent clinical testing procedures, leading to inadequate reporting. Second, because there were fewer members of high-risk groups (HIV patients, children, etc.) in the included articles, we did not conduct a subgroup analysis of high-risk groups, which may have led to an absence of specific diagnostic guidance for high-risk groups. Third, we have to admit that there was no search of conference proceedings or other grey documents (such as dissertations, etc.), which may affect the results of the systematic review. Finally, the results of the Deeks’ funnel-plot analysis suggested that publication bias fluctuated after combined diagnosis, appearing or increasing. Although some studies have pointed out that it is not appropriate to use funnel plots to evaluate publication bias in terms of diagnostic accuracy [19], we conducted a preliminary funnel-plot evaluation and, interestingly, found that the TST add-on TBST or IGRA add-on TBST might provide improved accuracy of TB infection diagnosis and contribute to more effective prevention and control of TB. However, we also considered that an “add-on” test might add to additional costs and the complexity of implementation. Based on the above limitations, we hope to optimize our research program in the future. We will select a population with the same basic characteristics and the same gold standard for the next study and try to avoid the impact of differences in diseases that affect different groups. In addition, we will expand the search to obtain more high-quality articles and conduct subgroup analyses for high-risk populations, which could provide more valuable guidance for the diagnosis of TB infection in this population and facilitate achieving the ultimate goal of eradicating TB.

## 6. Conclusions

In conclusion, among the limited articles we included, the new TB infection diagnostic test TBST appears to provide greater specificity. Interestingly, under certain conditions, the TST add-on TBST or IGRA add-on TBST exhibit better diagnostic accuracy than the TST or IGRA alone and can also provide more effective guidance for clinical practice. It is worth noting that further research is needed to investigate the sources of heterogeneity and continuously track and include more DTA articles related to the TBST in order to gain a deeper understanding of the utility and optimal implementation of these tests.

## Figures and Tables

**Table 1:** Characteristics of the included studies.

Study Authors	Type of Study	Country	Gold/Reference Standard	Index Test	Sample Size	Average Age (Years) *	BCG Scar (%)	Study Population
Wang et al. (2013) [20]	Prospective	China	Culture	IGRA	36	TB: 37.8 Control: 33.5	NA	Active TB
Iwagaitsu et al. (2016) [21]	Prospective	Japan	Clinical diagnosis	IGRA	68	TB: 71.6 Control: 56.6	100	Rheumatoid arthritis
Abdel-Samea et al. (2013) [22]	Prospective	Egypt	Culture	IGRA	50	28.2	20	TB
Jafari et al. (2013) [23]	Retrospective	Germany	Clinical diagnosis; culture	IGRA	96	TB: 54.7 Control: 57.2	NA	TB
Al Jahdali et al. (2013) [24]	Prospective	Saudi Arabia	Clinical diagnosis	IGRA	215	62.27	14.5	TB
Lee et al. (2013) [25]	Prospective	Korea	Clinical diagnosis	IGRA	89	TB: 61 Control: 66	NA	Pulmonary TB or TB pleurisy
Ferreira et al. (2015) [26]	Prospective	Brazil	QFT-GIT	TST (≥5 mm)	60	37	86.7	TB contacts
Ramadan et al. (2017) [27]	Prospective	Egypt	Clinical diagnosis	IGRA	40	TB: 28.3 Control: 48.4	NA	TB
Cirak et al. (2012) [28]	Prospective	Turkey	Culture	IGRA	100	57.3	NA	TB
Jia et al. (2016) [29]	Prospective	China	Clinical diagnosis	IGRA	365	TB: 29 Control: 59	NA	Suspected TB
Kamiya et al. (2013) [30]	Retrospective	Japan	Clinical diagnosis	IGRA	65	54	NA	TB
Shin, J. A. et al. (2015) [31]	Retrospective	Korea	Clinical diagnose	IGRA	418	45.5	NA	Suspected extrapulmonary -TB
Jeon et al. (2017) [32]	Retrospective	Korea	Clinical diagnosis	IGRA	567	TB: 52 Control: 61.4	NA	TB
Eldin et al. (2012) [33]	Prospective	Egypt	Clinical diagnosis	IGRA	38	48	NA	Suspected TB pleural effusion
Cheng et al. (2016) [34]	Retrospective	China	Culture	IGRA	20	TB: 41 Control: 47	NA	Suspected articular TB
Kim et al. (2016) [35]	Prospective	Korea	Clinical diagnosis	IGRA	244	TB: 33.7 Control: 35.5	NA	Suspected TB lymphadenitis
Li et al. (2017) [36]	Prospective	China	Culture	IGRA	300	28.47–44.29	100	TB
Liao et al. (2014) [37]	Prospective	China	Clinical diagnosis	IGRA	332	34.1–52.9	NA	TB
Gao et al. (2012) [38]	Prospective	China	Clinical diagnosis	IGRA	78	49	56	TB
Llorenc et al. (2013) [39]	Prospective	Spain	Clinical diagnosis	IGRA; TST (≥10 mm)	103	45.6	NA	TB-related uveitis
Jenum et al. (2014) [40]	Prospective	India	Clinical diagnosis	TST (≥10 mm)	705	14.8	NA	Children
Li et al. (2015) [41]	Prospective	China	Clinical diagnosis	IGRA	153	40–57.3	NA	Children
Feng et al. (2012) [42]	Prospective	China	Clinical diagnosis; culture	IGRA	182	52	100	HIV-negative; suspected active TB
Liu et al. (2013) [43]	Prospective	China	Clinical diagnosis; culture	IGRA	98	TB: 39 Control: 57	TB:5 Control:4	TB
Kussen et al. (2016) [44]	Prospective	Brazil	TST ≥ 5 mm	IGRA	140	38.9–52.3	78	HIV
Meyssonnier et al. (2012) [45]	Prospective	France	Clinical diagnosis; culture	IGRA	245	48	NA	Suspected active TB
Sun et al. (2015) [46]	Prospective	China	Clinical diagnosis	IGRA; TST (≥5 mm)	530	NA	91.7	Children
Chen et al. (2016) [47]	Retrospective	China	Clinical diagnosis	IGRA	26	TB: 27.2 Control: 30	NA	Pregnant women
Khawcharoenporn et al. (2015) [48]	Prospective	Thailand	QFT-GIT	TST (≥5 mm)	150	40	73	HIV-infected adults
Li et al. (2012) [49]	Prospective	China	Clinical diagnosis; culture	IGRA; TST (≥5 mm)	102	TB: 46.9 Control: 46.2	TB:33.3 Control:47.9	Suspected active PTB
Lei et al. (2013) [50]	Prospective	China	Clinical diagnosis; culture	IGRA; TST (≥10 mm)	191	TB: 37 Control: 36.2	NA	Intestinal TB and Crohn’s disease
Shi et al. (2016) [51]	Prospective	China	Clinical diagnosis; culture	IGRA	387	46	NA	Fever
Liu et al. (2016) [52]	Prospective	China	Clinical diagnosis	IGRA	65	TB: 34.9 Control: 38	NA	Female genital TB
El Azbaoui et al. (2016) [53]	Prospective	Morocco	Clinical diagnosis; culture	TST (≥10 mm)	109	7.8	100	Children
Park et al. (2016) [54]	Retrospective	Korea	Clinical diagnosis	IGRA; TST (≥10 mm)	224	TB: 46.5 Control: 56.6	NA	Sputum smear-negative PTB
Rumende et al. (2018) [55]	Prospective	Indonesia	Clinical diagnosis	IGRA	84	33.5	NA	Suspected extrapulmonary TB
Aggerbeck et al. (2018) [56]	Prospective	Denmark	Culture	TBST	1003	17	74.12	Children with TB; adults with HIV
Starshinova et al. (2018) [57]	Prospective	Russia	Clinical diagnosis	TBST	694	NA	100	Children and adults
Ruhwald et al. (2017) [58]	Prospective	Spain	Clinical diagnosis	TBST	977	NA	37.47	Volunteers
Bellete et al. (2002) [59]	Prospective	USA	Culture; clinical diagnosis	IGRA; TST (≥5 mm)	428	NA	31.8	TB screening
Detjen et al. (2007) [60]	Prospective	Germany	Culture; clinical diagnosis	IGRA	73	0.4–15	NA	TB; lymphadenitis
Mazurek et al. (2007) [61]	Prospective	USA	Culture; clinical diagnosis	IGRA; TST (≥5 mm)	148	46.6	33.8	TB
Kik et al. (2010) [62]	Prospective	Holland	Culture; clinical diagnosis	IGRA; TST (≥10 mm)	339	NA	80.8	TB
Lee et al. (2011) [63]	Prospective	Korea	Culture; clinical diagnosis	IGRA; TST	143	20–29	71.3	Suspected TB
Ates et al. (2011) [64]	Prospective	Turkey	Clinical diagnosis	IGRA	72	NA	54	Pleural TB
Sun et al. (2010) [65]	Prospective	China	Clinical diagnosis	IGRA; TST (≥10 mm)	125	NA	77.6	Children with active TB
Syed Ahamed Kabeer et al. (2010) [66]	Prospective	India	Culture; clinical diagnosis	IGRA; TST (≥10 mm)	277	37	NA	Adult with pulmonary
Hoff et al. (2016) [67]	Prospective	South Africa	Clinical diagnosis	TBST	297	18–65	12.6	Active TB adults
Aggerbeck et al. (2013) [68]	Prospective	Italy	Culture	TBST	151	NA	NA	Active TB adults

TB, tuberculosis; NA, not available; (years) *, average age is either mean or median (range); IGRA, interferon-gamma release assay; TST, tuberculin skin test; TBST, *Mycobacterium tuberculosis* antigen-based skin test; QFT-GIT, QuantiFERON^®^-TB Gold In-Tube; BCG, bacille Calmette–Guerin; USA, United States of America; ROC, receiver operating characteristic; HIV, human immunodeficiency virus.

**Table 2:** Assessment of the risk of bias in the included studies.

Study	Patient Selection	Index Test	Reference Standard	Flow and Timing	Overall
Wang et al. [20]	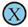	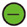	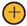	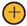	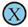
Iwagaitsu et al. [21]	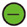	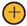	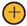	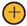	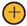
Abdel-Samea et al. [22]	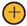	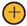	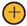	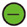	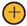
Jafari et al. [23]	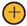	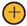	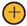	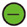	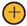
Al Jahdali et al. [24]	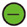	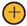	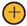	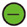	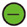
Lee et al. [25]	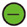	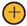	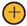	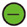	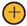
Ferreira et al. [26]	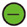	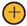	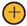	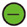	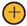
Ramadan et al. [27]	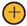	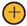	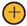	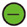	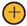
Cirak et al. [28]	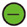	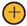	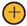	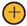	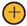
Jia et al. [29]	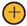	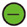	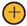	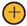	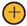
Kamiya et al. [30]	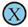	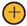	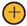	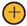	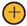
Shin et al. [31]	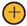	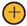	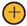	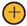	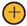
Jeon et al. [32]	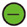	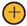	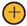	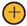	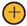
Eldin et al. [33]	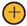	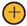	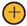	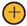	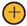
Cheng et al. [34]	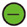	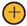	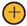	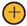	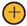
Kim et al. [35]	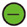	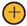	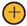	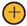	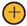
Li et al. [36]	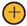	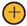	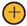	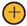	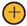
Liao et al. [37]	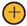	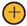	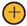	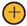	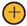
Gao et al. [38]	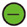	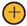	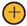	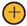	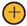
Llorenc et al. [39]	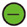	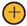	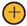	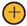	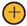
Jenum et al. [40]	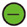	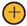	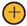	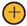	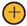
Li et al. [41]	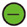	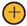	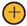	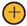	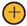
Feng et al. [42]	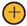	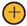	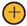	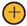	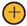
Liu et al. [43]	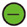	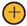	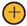	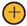	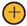
Kussen et al. [44]	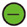	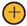	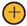	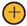	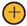
Meyssonnier et al. [45]	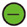	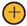	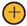	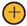	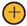
Sun et al. [46]	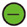	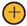	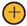	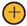	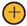
Chen et al. [47]	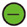	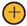	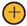	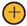	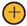
Khawcharoenporn et al. [48]	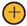	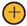	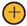	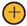	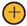
Li et al. [49]	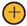	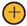	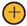	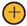	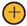
Lei et al. [50]	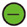	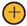	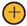	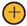	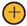
Shi et al. [51]	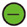	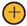	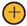	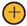	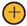
Liu et al. [52]	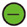	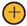	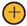	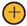	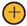
El Azbaoui et al. [53]	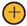	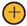	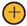	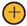	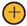
Park et al. [54]	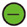	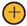	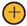	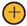	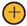
Rumende et al. [55]	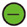	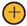	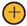	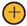	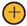
Aggerbeck et al. [56]	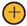	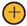	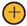	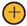	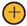
Starshinova et al. [57]	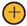	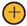	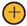	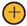	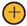
Ruhwald et al. [58]	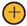	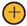	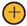	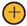	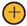
Bellete et al. [59]	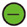	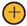	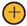	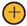	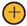
Detjen et al. [60]	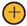	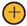	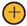	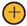	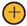
Mazurek et al. [61]	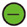	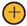	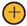	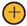	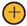
Kik et al. [62]	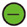	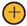	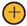	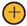	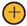
Lee et al. [63]	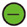	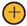	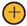	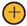	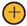
Ates et al. [64]	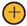	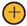	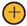	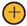	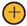
Lin et al. [65]	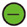	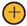	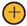	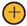	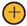
Ahamed Kabeer et al. [66]	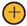	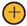	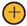	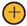	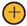
Hoff et al. [67]	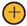	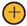	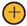	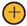	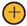
Aggerbeck et al. [68]	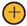	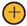	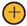	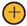	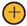

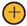
 Low risk 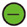 Unclear risk 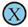 High risk.

## Data Availability

All data used in the study are included in the manuscript and the Supplementary Materials.

## Supplementary Materials

The following supporting information can be downloaded at: https://www.mdpi.com/article/10.3390/pathogens13121050/s1, Figure S1: Forest plot for sensitivity in IGRA. TP, true positive; FN, false negative [20,21,22,23,24,25,27,28,29,30,31,32,33,34,35,36,37,38,39,41,42,43,44,45,46,47,48,49,50,51,52,54,55,59,60,61,62,63,64,65,66]; Figure S2: Forest plot for specificity in IGRA. TN, true negative; FP, false positive [20,21,22,23,24,25,27,28,29,30,31,32,33,34,35,36,37,38,39,41,42,43,44,45,46,47,48,49,50,51,52,54,55,59,60,61,62,63,64,65,66]; Figure S3: Forest plot for sensitivity and specificity in TST (A, sensitivity, B, specificity). TP, true positive; FN, false negative; TN, true negative; FP, false positive [26,39,40,46,48,49,50,53,59,60,61,62,63,65,66]; Figure S4: Forest plot for sensitivity and specificity in TBST (A, sensitivity [56,57,58,67,68]; B, specificity [58,67,68].) TP, true positive; FN, false negative; TN, true negative; FP, false positive. Figure S5: Summary receiver operating curve (SROC) of the TST-BCG (A), the TST add-on TBST (B); and the Deeks’ funnel plot in TST-BCG (C) and the TST add-on TBST (D); Figure S6: Fagan nomogram in TST-BCG (A) and TST add-on TBST (B); Figure S7: SROC curves and Deeks’ funnel plots for different methods. SROC curves for he IGRA (A) and TST (B), and Deeks’ funnel plots for the IGRA (C) and TST (D); Figure S8: Table S1: The search strategy in each database and results; Fagan nomogram for different methods. The IGRA (A) and TST (B); Figure S9: Summary receiver operating curve (SROC) of the IGRA-BCG (A), the IGRA add-on TBST (B); and the Deeks’ funnel plot in IGRA-BCG (C) and the IGRA add-on TBST (D); Figure S10: Fagan nomogram in IGRA-BCG (A) and IGRA add-on TBST (B); Figure S11: Risk of bias presented as percentages across included studies; Table S2: Consolidated statistics regarding the included studies; Table S3: Abbreviations and acronyms.
