# Supplementary material for: Diagnostic Accuracy of Mycobacterium tuberculosis Antigen-Based Skin Tests (TBSTs) for Tuberculosis Infection Compared with TST and IGRA: A Network Meta-Analysis
Source: Pathogens. 2024 Nov 29;13(12):1050. doi: 10.3390/pathogens13121050 (PMC11728611; doi:10.3390/pathogens13121050)
Supplement: Supplementary file 1 [file pathogens-13-01050-s001.zip › pathogens-3294481-Supplementary materials.pdf]

Supplementary Table S1. The search strategy in each database and results.

Database	Search strategy	Date	Result
PubMed	(((("C-Tb"[Title/Abstract] OR "Cy-Tb"[Title/Abstract] OR "Diaskintest"[Title/Abstract] OR "C-TST"[Title/Abstract]) AND "english"[Language]) OR (("tuberculin test"[Title/Abstract] OR "test tuberculin"[Title/Abstract] OR "tests tuberculin"[Title/Abstract] OR "Tuberculin Tests"[Title/Abstract]) AND "english"[Language]) OR (("interferon gamma release tests"[Title/Abstract] OR "interferon gamma release tests"[Title/Abstract] OR "Interferon-gamma Release Test"[Title/Abstract] OR "release test interferon gamma"[Title/Abstract] OR "release tests interferon gamma"[Title/Abstract] OR "test interferon gamma release"[Title/Abstract] OR "tests interferon gamma release"[Title/Abstract] OR "interferon gamma release assays"[Title/Abstract] OR "interferon gamma release assays"[Title/Abstract] OR "interferon gamma release assay"[Title/Abstract] OR "assay interferon gamma release"[Title/Abstract] OR "assays interferon gamma release"[Title/Abstract] OR "interferon gamma release assay"[Title/Abstract] OR "release assay interferon gamma"[Title/Abstract] OR ("interferon gamma release tests"[MeSH Terms] OR ("interferon gamma"[Title/Abstract] AND "release"[Title/Abstract] AND "tests"[Title/Abstract]) OR "interferon gamma release tests"[Title/Abstract] OR ("release"[Title/Abstract] AND "assays"[Title/Abstract] AND "interferon"[Title/Abstract] AND "gamma"[Title/Abstract]))) AND "english"[Language])) AND "english"[Language] AND (("Tuberculosis"[Title/Abstract] OR "Tuberculoses"[Title/Abstract] OR "Koch's Disease"[Title/Abstract] OR "Koch Disease"[Title/Abstract] OR "Mycobacterium tuberculosis Infection"[Title/Abstract] OR "infection mycobacterium tuberculosis"[Title/Abstract] OR "infections mycobacterium tuberculosis"[Title/Abstract] OR "Mycobacterium tuberculosis Infections"[Title/Abstract] OR "Mycobacterium tuberculosis"[Title/Abstract] OR "Mycobacterium tuberculosis H37Rv"[Title/Abstract]) AND "english"[Language]) AND (("diagnosable"[Title/Abstract] OR "diagnosi"[Title/Abstract] OR "diagnosis"[MeSH Terms] OR "diagnosis"[Title/Abstract] OR "diagnose"[Title/Abstract] OR "diagnosed"[Title/Abstract] OR "diagnoses"[Title/Abstract] OR "diagnosing"[Title/Abstract] OR "diagnosis"[MeSH Subheading] OR "diagnosable"[Title/Abstract] OR "diagnosi"[Title/Abstract] OR "diagnosis"[MeSH Terms] OR "diagnosis"[Title/Abstract] OR "diagnose"[Title/Abstract] OR "diagnosed"[Title/Abstract] OR "diagnoses"[Title/Abstract] OR "diagnosing"[Title/Abstract] OR "diagnosis"[MeSH Subheading] OR "Diagnoses and Examinations"[Title/Abstract] OR "Examinations and Diagnoses"[Title/Abstract] OR "Diagnoses and Examination"[Title/Abstract] OR "Examination and Diagnoses"[Title/Abstract] OR "Postmortem Diagnosis"	2024/9/30	3383

	[Title/Abstract] OR "diagnoses postmortem"[Title/Abstract] OR "diagnosis postmortem"[Title/Abstract] OR "Postmortem Diagnoses"[Title/Abstract] OR "Antemortem Diagnosis"[Title/Abstract] OR "Antemortem Diagnoses"[Title/Abstract] OR "diagnoses antemortem"[Title/Abstract] OR "diagnosis antemortem"[Title/Abstract]) AND "english"[Language])) AND (english[Filter])		
Embase	1 (C-Tb or Cy-Tb or Diaskintest or C-TST or tuberculin test or Test, Tuberculin or Tests, Tuberculin or Interferon-gamma Release Tests or Interferon gamma Release Tests or Interferon-gamma Release Test or Release Test, Interferon-gamma or Release Tests, Interferon-gamma or Test, Interferon-gamma Release or Tests, Interferon-gamma Release or Interferon-gamma Release Assays or Interferon gamma Release Assays or Interferon-gamma Release Assay or Assay, Interferon-gamma Release or Assays, Interferon-gamma Release or Interferon gamma Release Assay or Release Assay, Interferon-gamma or Release Assays, Interferon-gamma).ab.<br>2 (C-Tb or Cy-Tb or Diaskintest or C-TST or tuberculin test or Test, Tuberculin or Tests, Tuberculin or Interferon-gamma Release Tests or Interferon gamma Release Tests or Interferon-gamma Release Test or Release Test, Interferon-gamma or Release Tests, Interferon-gamma or Test, Interferon-gamma Release or Tests, Interferon-gamma Release or Interferon-gamma Release Assays or Interferon gamma Release Assays or Interferon-gamma Release Assay or Assay, Interferon-gamma Release or Assays, Interferon-gamma Release or Interferon gamma Release Assay or Release Assay, Interferon-gamma or Release Assays, Interferon-gamma).ti.<br>3 (Mycobacterium tuberculosis or Mycobacterium tuberculosis H37Rv or Tuberculosis or Tuberculoses or Kochs Disease or Koch's Disease or Koch Disease or Mycobacterium tuberculosis Infection or Infection, Mycobacterium tuberculosis or Infections, Mycobacterium tuberculosis or Mycobacterium tuberculosis Infections).ab.<br>4 (Mycobacterium tuberculosis or Mycobacterium tuberculosis H37Rv or Tuberculosis or Tuberculoses or Kochs Disease or Koch's Disease or Koch Disease or Mycobacterium tuberculosis Infection or Infection, Mycobacterium tuberculosis or Infections, Mycobacterium tuberculosis or Mycobacterium tuberculosis Infections).ti.<br>5 (Diagnosis or Diagnoses or Diagnose or (Diagnoses and Examinations) or (Examinations and Diagnoses) or (Diagnoses and Examination) or (Examination and Diagnoses) or Postmortem Diagnosis or Diagnoses, Postmortem or Diagnosis,	2024/9/30	1965

	Postmortem or Postmortem Diagnoses or Antemortem Diagnosis or Antemortem Diagnoses or Diagnoses, Antemortem or Diagnosis, Antemortem).ab.<br>6 (Diagnosis or Diagnoses or Diagnose or (Diagnoses and Examinations) or (Examinations and Diagnoses) or (Diagnoses and Examination) or (Examination and Diagnoses) or Postmortem Diagnosis or Diagnoses, Postmortem or Diagnosis, Postmortem or Postmortem Diagnoses or Antemortem Diagnosis or Antemortem Diagnoses or Diagnoses, Antemortem or Diagnosis, Antemortem).ti.<br>(1 or 2) and (3 or 4) and (4 or 5)		
Cochrane	(((C-Tb):ti,ab,kw OR (Cy-Tb):ti,ab,kw OR (Diaskintest):ti,ab,kw OR (C-TST):ti,ab,kw) OR (("tuberculin test"):ti,ab,kw OR ("Test, Tuberculin"):ti,ab,kw OR ("Tests, Tuberculin"):ti,ab,kw) OR (("Interferon-gamma Release Tests"):ti,ab,kw OR ("Interferon gamma Release Tests"):ti,ab,kw OR ("Interferon-gamma Release Test"):ti,ab,kw OR ("Release Test, Interferon-gamma"):ti,ab,kw OR ("Release Tests, Interferon-gamma"):ti,ab,kw OR ("Test, Interferon-gamma Release"):ti,ab,kw OR ("Tests, Interferon-gamma Release"):ti,ab,kw OR ("Interferon-gamma Release Assays"):ti,ab,kw OR ("Interferon gamma Release Assays"):ti,ab,kw OR ("Interferon-gamma Release Assay"):ti,ab,kw OR ("Assay, Interferon-gamma Release"):ti,ab,kw OR ("Assays, Interferon-gamma Release"):ti,ab,kw OR ("Interferon gamma Release Assay"):ti,ab,kw OR ("Release Assay, Interferon-gamma"):ti,ab,kw OR ("Release Assays, Interferon-gamma"):ti,ab,kw)) AND ((Diagnosis):ti,ab,kw OR (Diagnoses):ti,ab,kw OR (Diagnose):ti,ab,kw OR ("Diagnoses and Examinations"):ti,ab,kw OR ("Examinations and Diagnoses"):ti,ab,kw OR ("Diagnoses and Examination"):ti,ab,kw OR ("Examination and Diagnoses"):ti,ab,kw OR ("Postmortem Diagnosis"):ti,ab,kw OR ("Diagnoses, Postmortem"):ti,ab,kw OR ("Diagnosis, Postmortem"):ti,ab,kw OR ("Postmortem Diagnoses"):ti,ab,kw OR ("Antemortem Diagnosis"):ti,ab,kw OR ("Antemortem Diagnoses"):ti,ab,kw OR ("Diagnoses, Antemortem"):ti,ab,kw OR ("Diagnosis, Antemortem"):ti,ab,kw) AND ((“Mycobacterium tuberculosis”):ti,ab,kw OR (“Mycobacterium tuberculosis H37Rv”):ti,ab,kw OR (Tuberculosis):ti,ab,kw OR (Tuberculoses):ti,ab,kw OR (“Kochs Disease”):ti,ab,kw OR (“Koch's Disease”):ti,ab,kw OR (“Koch Disease”):ti,ab,kw OR (“Mycobacterium tuberculosis Infection”):ti,ab,kw OR ("Infection, Mycobacterium tuberculosis"):ti,ab,kw OR ("Infections, Mycobacterium tuberculosis"):ti,ab,kw OR ("Mycobacterium tuberculosis Infections"):ti,ab,kw)	2024/9/30	174

Supplementary Table S2. Consolidated statistics regarding the included studies.

**A**	**Total population**		**vs**	**BCG-vaccinated population**	
	Estimate	95% CI		Estimate	95% CI
Sensitivity	0.82	(0.77, 0.85)	Sensitivity	0.8	(0.72, 0.86)
Specificity	0.84	(0.78, 0.88)	Specificity	0.87	(0.77, 0.92)
PLR	5.1	(3.7, 6.8)	*PLR*	5.9	(3.4, 10.2)
NLR	0.22	(0.17, 0.28)	*NLR*	0.23	(0.17, 0.33)
DOR	23	(15, 36)	*DOR*	25	(12, 51)

**B**	**IGRA**		**vs**	**TST**	
	Estimate	95% CI		Estimate	95% CI
Sensitivity	0.83	(0.78, 0.87)	Sensitivity	0.78	(0.64, 0.87)
Specificity	0.83	(0.78, 0.88)	Specificity	0.75	(0.6, 0.86)
PLR	5	(3.7, 6.9)	*PLR*	3.1	(1.9, 5.2)
NLR	0.2	(0.15, 0.26)	*NLR*	0.3	(0.18, 0.49)
DOR	25	(15, 42)	*DOR*	11	(5, 23)

**C**	**TST-BCG**		**vs**	**TST add-on TBST**	
	Estimate	95% CI		Estimate	95% CI
Sensitivity	0.77	(0.62, 0.88)	Sensitivity	0.76	(0.64, 0.84)
Specificity	0.74	(0.49, 0.90)	Specificity	0.84	(0.66, 0.96)
PLR	3	(1.3, 6.8)	*PLR*	5.9	(2.0, 17.1)
NLR	0.3	(0.17, 0.54)	*NLR*	0.28	(0.18, 0.42)
DOR	10	(3, 33)	*DOR*	21	(6, 74)

**D**	**IGRA-BCG**		**vs**	**IGRA add-on TBST**	
	Estimate	95% CI		Estimate	95% CI
Sensitivity	0.82	(0.71, 0.89)	Sensitivity	0.81	(0.71, 0.88)
Specificity	0.86	(0.75, 0.92)	Specificity	0.9	(0.81, 0.95)
PLR	5.7	(3.2, 10.0)	*PLR*	8	(4.2, 15.2)
NLR	0.21	(0.13, 0.35)	*NLR*	0.22	(0.14, 0.33)
DOR	26	(11, 61)	*DOR*	37	(16, 84)

PLR, positive likelihood ratio; *NLR*, negative likelihood ratio; *DOR*, diagnostic odds ratio; CI, confidence interval.

Supplementary Table S3. Abbreviations and acronyms.

**Abbreviations and acronyms**	
AUC	area under the curve
BCG	bacille Calmette-Guerin
CFP-10	culture filtrate protein 10
CI	confidence interval
DOR	diagnostic odds ratio
DTA	diagnostic test accuracy
ELISA	enzyme-linked immunosorbent assay
ESAT-6	early secretory antigenic target 6 kDa protein
FN	false negative
FP	false positive
HIV	human immunodeficiency virus
IGRA	interferon-gamma release assay
LR	likelihood ratio
Mtb	*Mycobacterium tuberculosis*
NLR	negative likelihood ratio
NMA	network meta-analysis
PICO	population, intervention, comparator and outcome
PLR	positive likelihood ratio
PRISMA	preferred reporting items for systematic reviews and meta-analyses
QFT-GIT	QuantiFERON-TB Gold In-Tube
QFT-Plus	QuantiFERON-TB Gold Plus
QUADAS-2	quality assessment of diagnostic accuracy studies tool
SROC	summarized receiver operating characteristic curve
TB	tuberculosis
TBST	*Mycobacterium tuberculosis* antigen-based skin test
TN	true negative
TP	true positive
T-SPOT. TB	Oxford Immunotec T-SPOT ®. *TB* assays
TST	tuberculin skin test
USA	United States of America

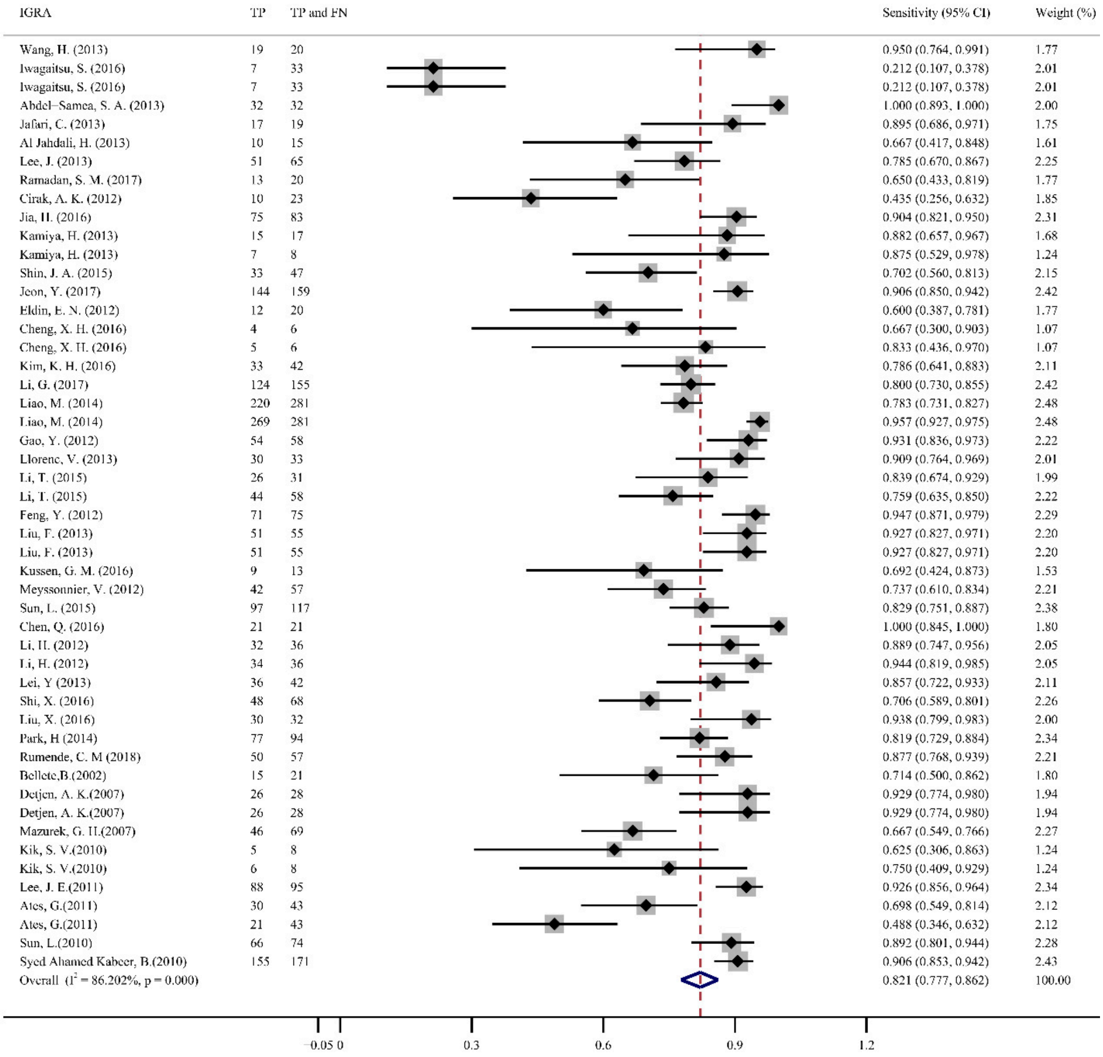

Supplementary Figure S1. Forest plot for sensitivity in IGRA. TP, true positive; FN, false negative [20-25,27-39,41-52,54,55,59-66].

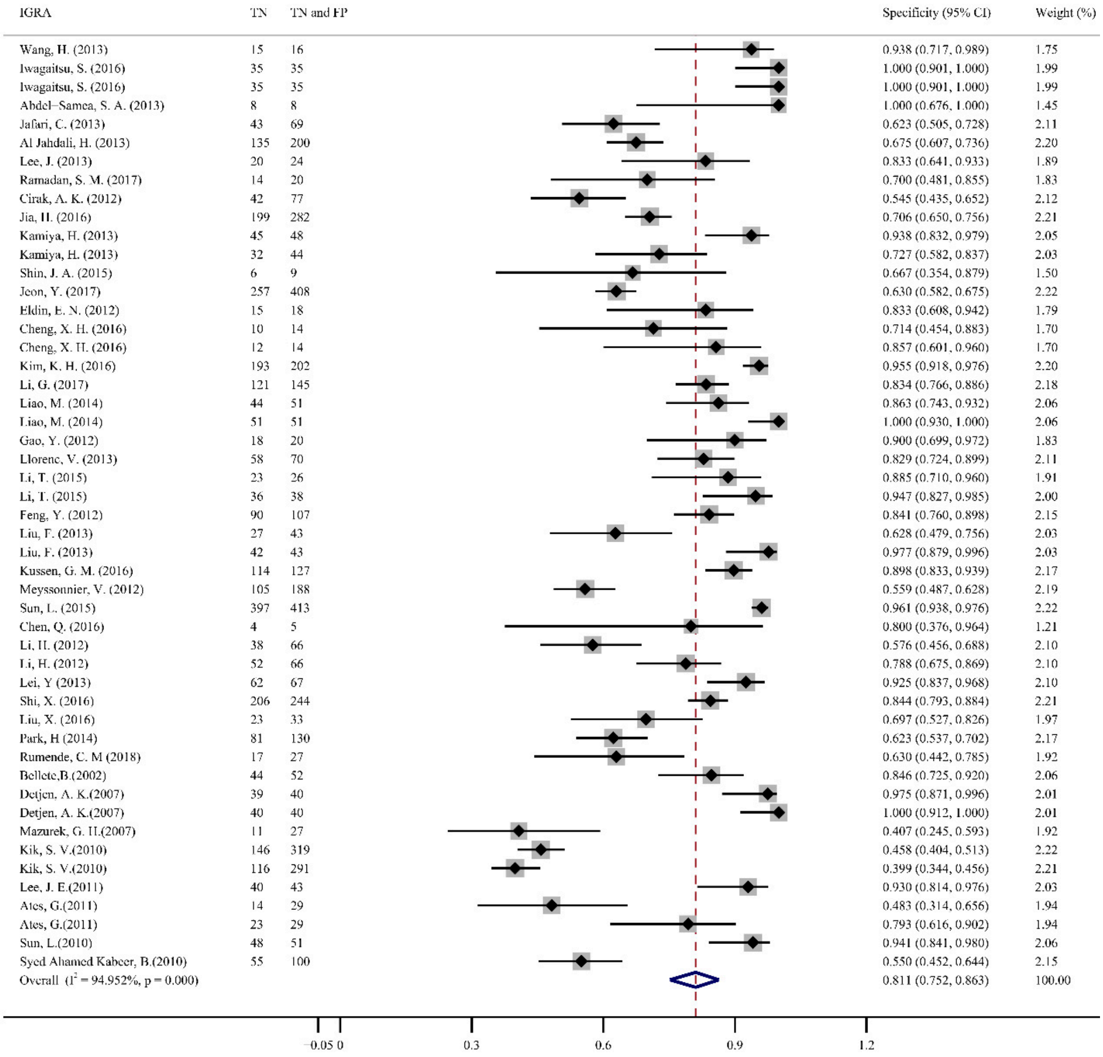

Supplementary Figure S2. Forest plot for specificity in IGRA. TN, true negative; FP, false positive[20-25,27-39,41-52,54,55,59-66].

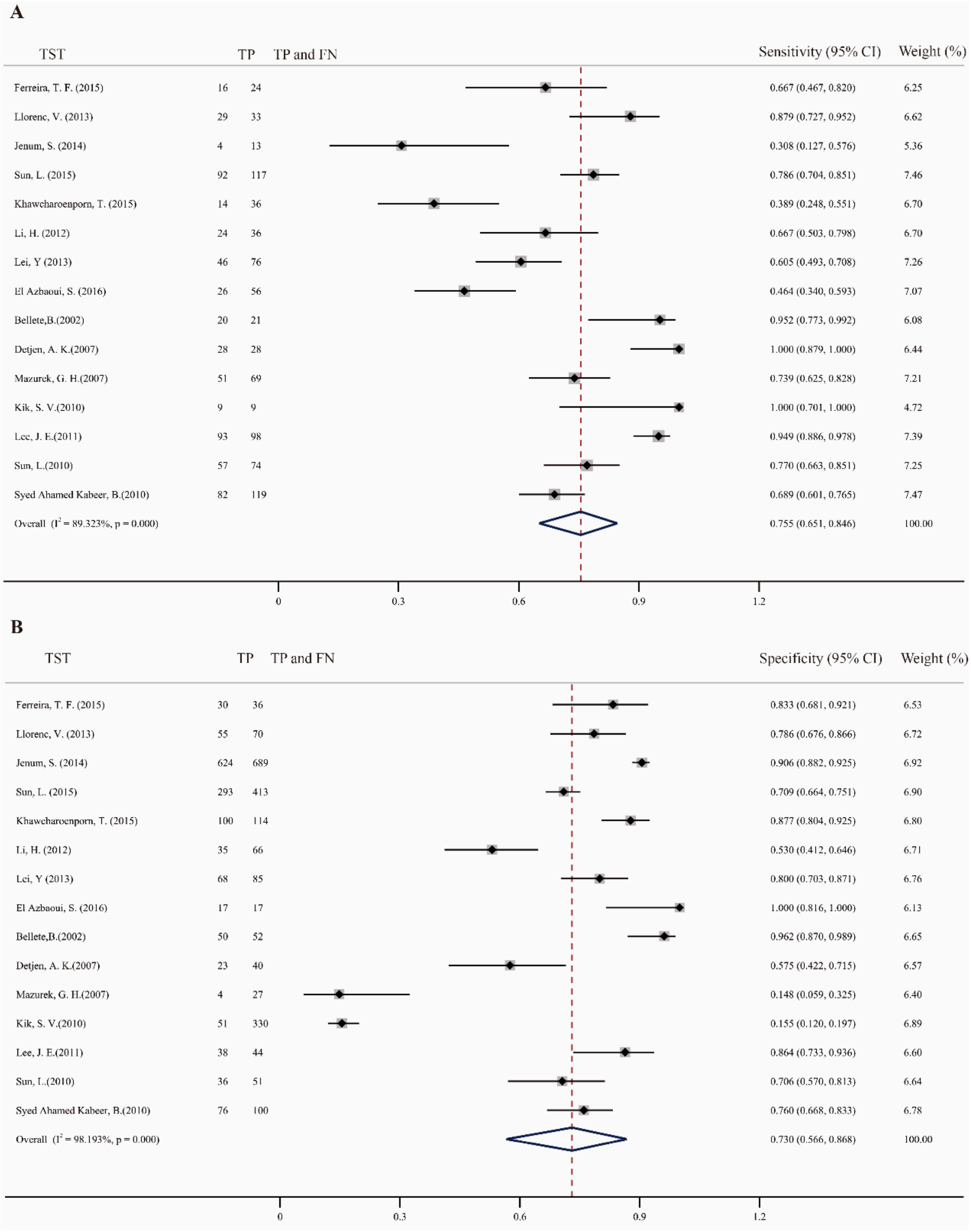

Supplementary Figure S3. Forest plot for sensitivity and specificity in TST (A, sensitivity, B, specificity). TP, true positive; FN, false negative; TN, true negative; FP, false positive[26,39,40,46,48-50,53,59-63,65,66].

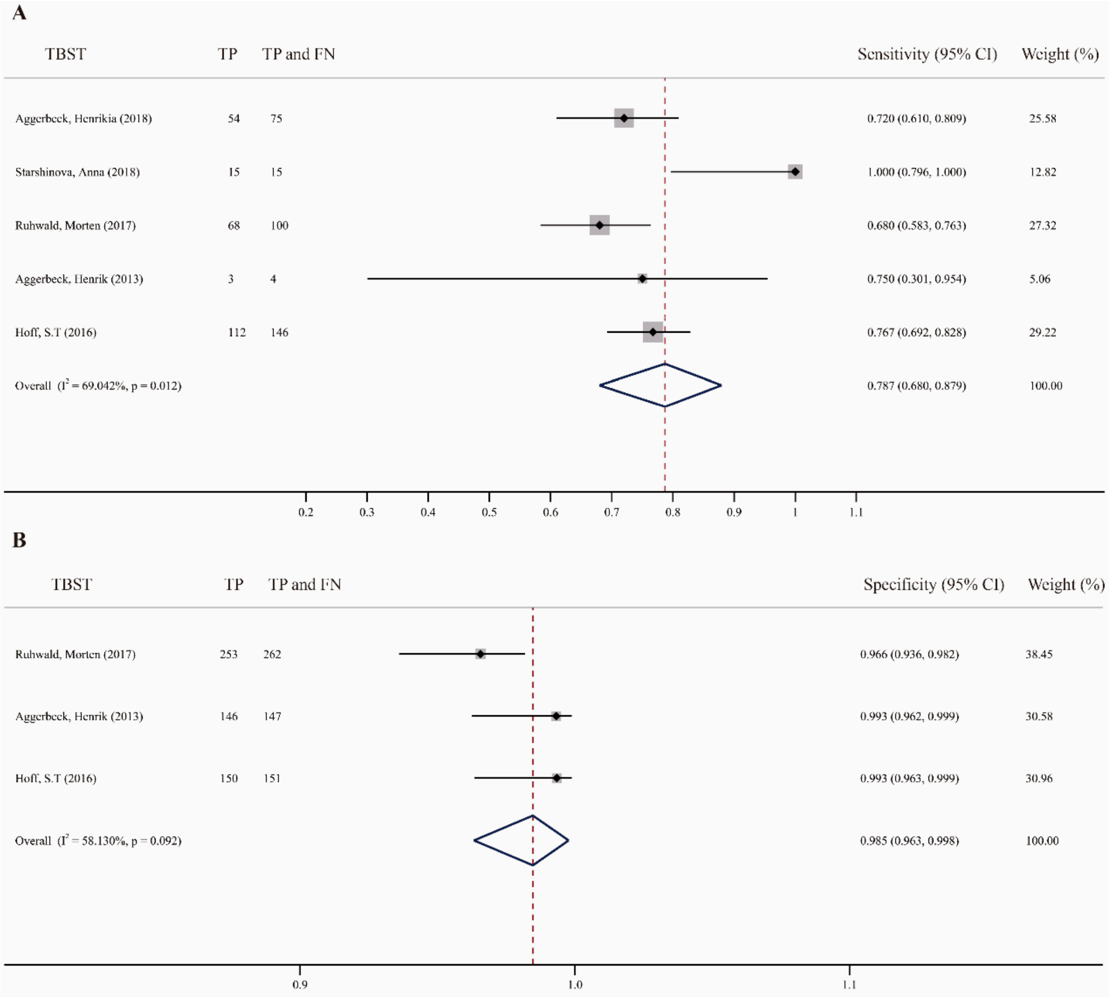

Supplementary Figure S4. Forest plot for sensitivity and specificity in TBST (A, sensitivity[56-58,67,68]; B, specificity[58,67,68].) TP, true positive; FN, false negative; TN, true negative; FP, false positive.

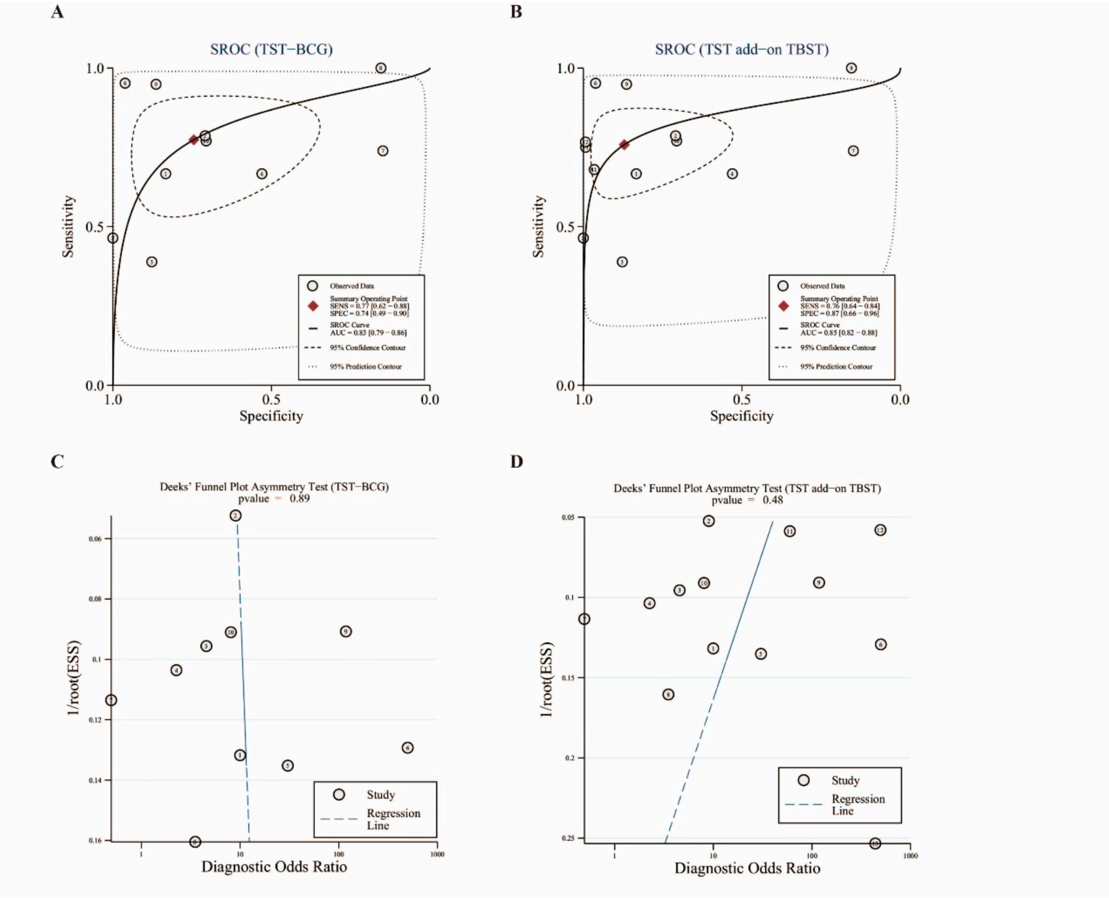

Supplementary Figure S5. Summary receiver operating curve (SROC) of the TST-BCG (A), the TST add-on TBST (B); and the Deeks' funnel plot in TST-BCG (C) and the TST add-on TBST (D).

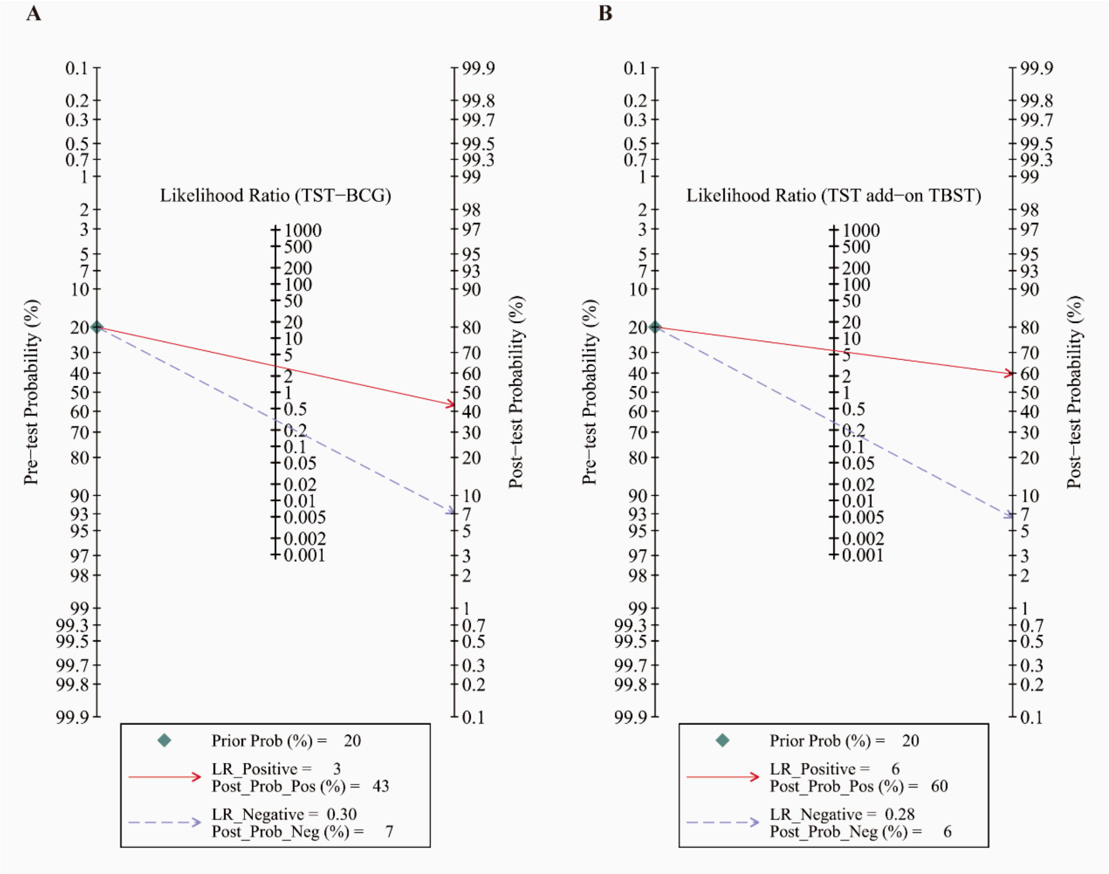

Supplementary Figure S6. Fagan nomogram in TST-BCG (A) and TST add-on TBST (B).

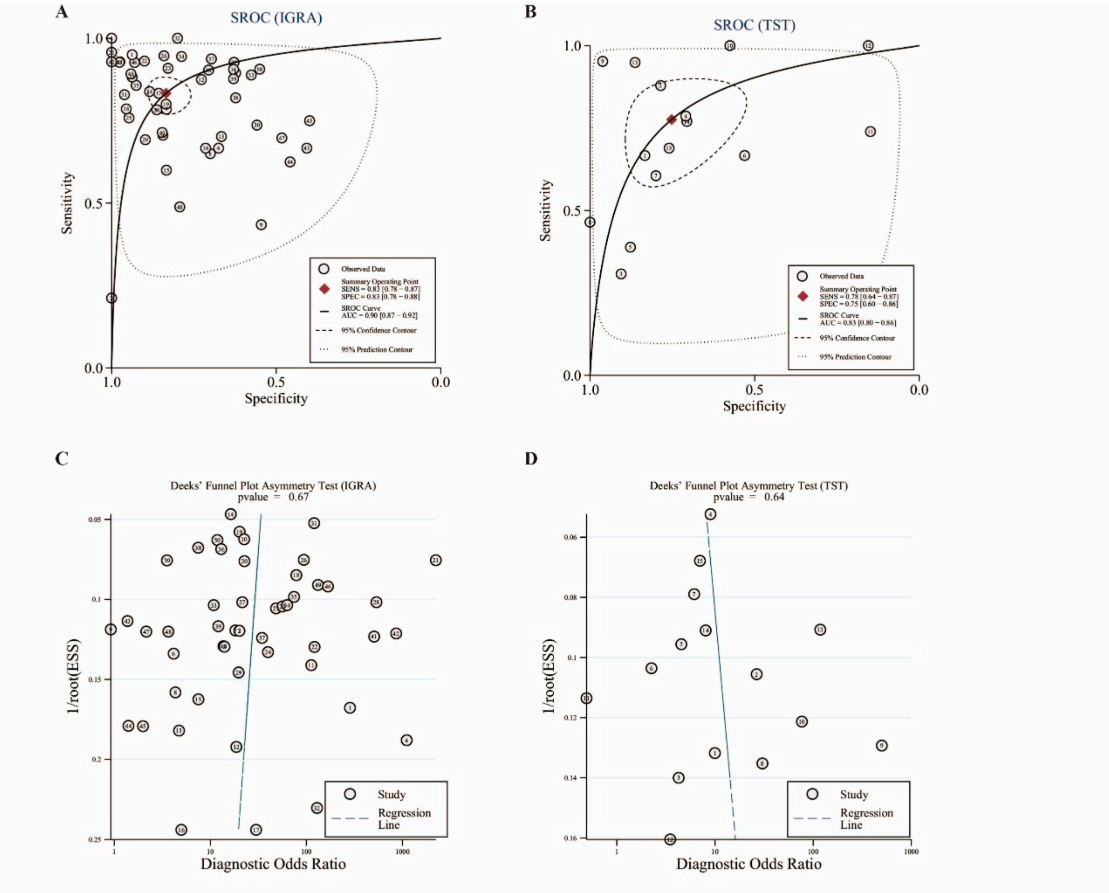

Supplementary Figure S7. SROC curves and Deeks' funnel plots for different methods. SROC curves for he IGRA (A) and TST (B), and Deeks' funnel plots for the IGRA (C) and TST (D).

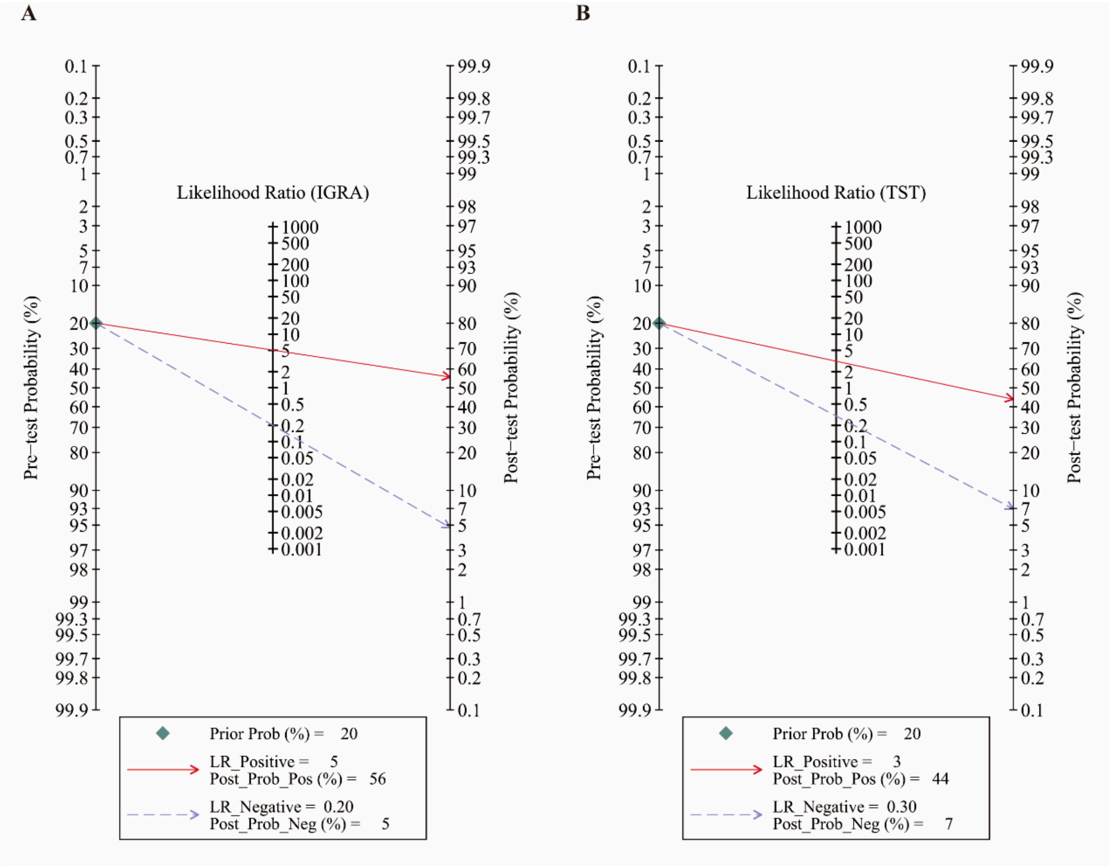

Supplementary Figure S8. Fagan nomogram for different methods. The IGRA (A) and TST (B).

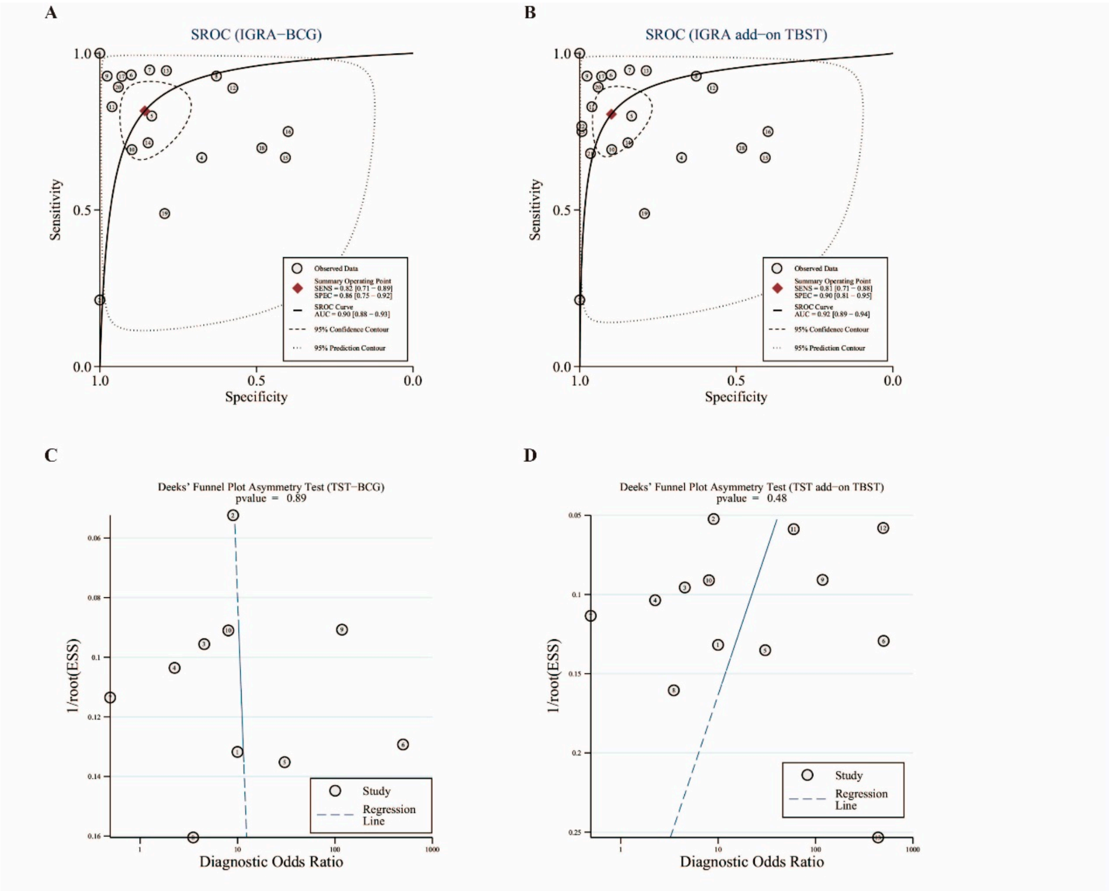

Supplementary Figure S9. Summary receiver operating curve (SROC) of the IGRA-BCG (A), the IGRA add-on TBST (B); and the Deeks' funnel plot in IGRA-BCG (C) and the IGRA add-on TBST (D).

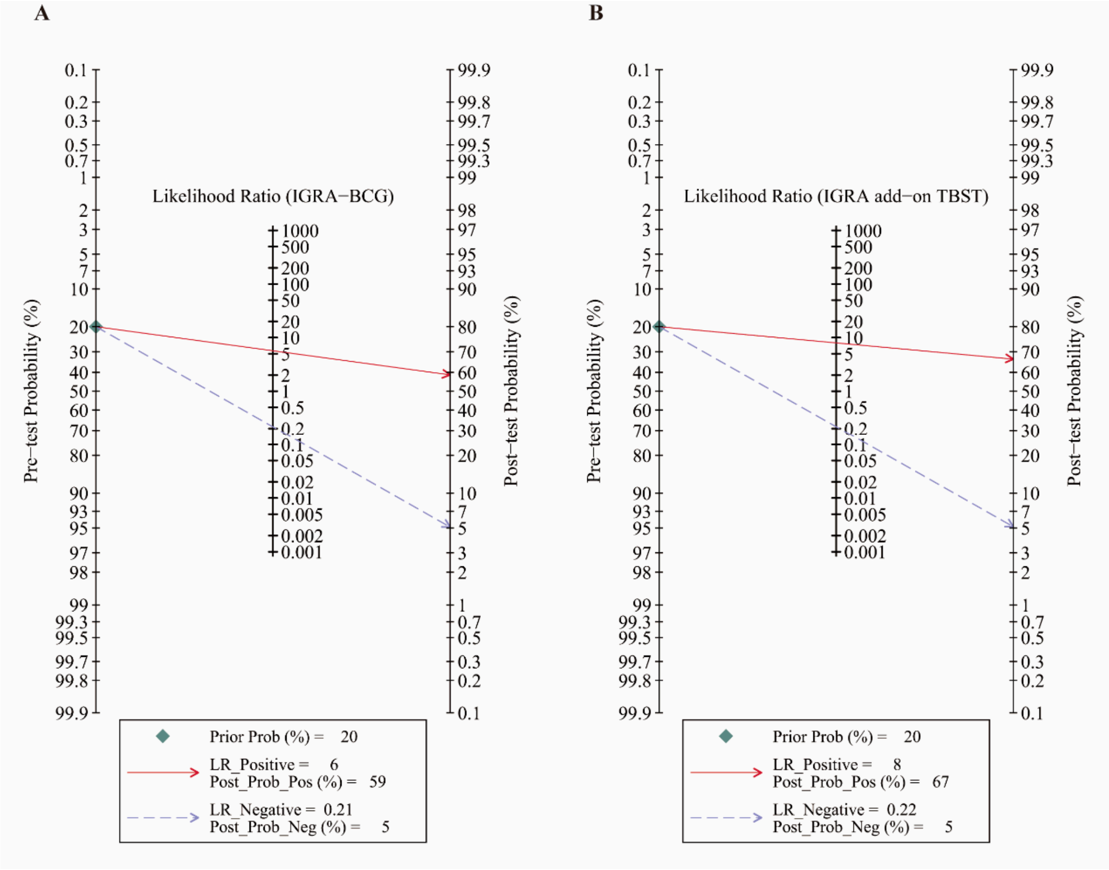

Supplementary Figure S10. Fagan nomogram in IGRA-BCG (A) and IGRA add-on TBST (B).

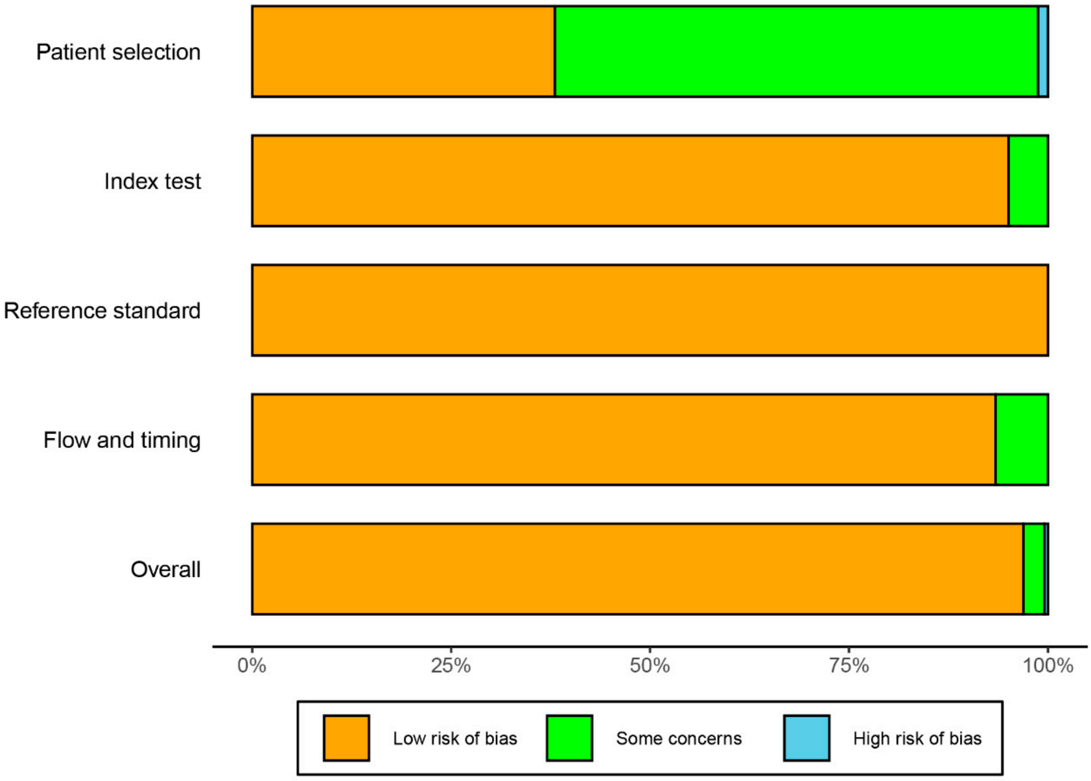

Supplementary Figure S11. Risk of bias presented as percentages across included studies.
